# Libyan cancer patients at King Hussein Cancer Center for more than a decade, the current situation, and a future vision

**DOI:** 10.3389/fonc.2022.1025757

**Published:** 2023-01-26

**Authors:** Madiha Erashdi, Abdallah Al-Ani, Asem Mansour, Maysa Al-Hussaini

**Affiliations:** ^1^ Department of Pathology, James Cook University Hospital, South Tees National Health Service (NHS) Foundation Trust, Middlesbrough, United Kingdom; ^2^ Office of Scientific Affairs and Research, King Hussein Cancer Center, Amman, Jordan; ^3^ Human Research Participants Protection Office, King Hussein Cancer Center, Amman, Jordan

**Keywords:** Libyan cancer patients, King Hussein Cancer Center, breast, gastrointestinal tract, hematolymphoid, awareness, screening programs

## Abstract

**Background:**

Since 2011, the Libyan civil war crisis had affected all dimensions of livelihood including cancer care. This has resulted in a steady incline in the number of Libyan patients with cancer seeking oncologic care and management in Tunisia, Egypt and Jordan, among others. King Hussein Cancer Center (KHCC) has been one of the main destinations for Libyan patients with cancer for more than a decade.

**Aim:**

We are reporting on the characteristics of Libyan patients with cancer presenting to KHCC during the past fourteen years.

**Methods:**

We performed a retrospective chart review of all Libyan patients with cancer presenting to KHCC between 2006 and 2019.

**Results:**

A total of 3170 records were included in the final analysis. The overall sample was predominantly adults (71%) with a male-to-female ratio of 1:1.2. Overall, the most common referred cancers to KHCC were breast (21%), hematolymphoid (HL) (17%), and gastrointestinal tract (GIT) (16.2%) cancers. Breast cancer was the most common among adult females (41.7%), GIT among adult males (23.6%), and HL among pediatrics (38.5%). Around 37.8% of patients presented with distant metastasis at their first encounter at KHCC, among which 14.7% were candidates for palliative care.

**Conclusion:**

The sustenance of treatment for Libyan patients with cancer requires extensive collaboration between governmental and private sectors. The Libyan oncological landscape could benefit from national screening and awareness programs, twining programs and telemedicine, introduction of multidisciplinary boards, and the formulation of a national cancer registry. Adopting the successful models at KHCC can help to augment the oncology services within the Libyan healthcare sector.

## 1 Introduction

Disparity in cancer care exists on a fundamental economic level as patients with cancer in low or middle-income regions face significantly worse survival outcomes compared to those within the developed world (1). Being the 2^nd^ most common cause of death after cardiovascular disease, the burden of cancer within low or middle-income countries is increasing (2). Such a burden is amplified by the presence of armed conflicts (e.g., wars, armed hostilities, etc.). These conflicts may increase cancer incidence, augment complications, and worsen survival outcomes as it disrupts care seeking and delivery in the short term among all aspects of oncological care (3, 4). Due to the changing political contexts and priorities during times of armed conflict, shifting of resources away from cancer care is expected. Other war-related determinants of cancer care continuity include forced migration, increased psychological burden, killing or fleeing of medical staff, and delivery of sub-optimal treatments (2). On the long term, war impacts cancer burden through intoxicating the environment or by encouraging unhealthy behaviors such as substance abuse (5, 6). The impact of armed conflict in war-torn areas is well documented within the literature. Prime examples of such an effect are Syria, Iraq, Afghanistan, and Gaza-Palestine (7–11). Patients from these areas often present with advanced stages and have high mortality rates owing to misdiagnosis, delays in diagnosis, lack of healthcare access, lack of essential treatment regimens, and interrupted treatment.

Over the past decade, Libya has witnessed two distinct civil wars which led to an ongoing conflict among rival factions seeking military and political control over the nation. Such conditions did not only subject Libyan patients with cancer to the aforementioned challenges of cancer care but also displaced them within the country and to neighboring, and sometimes distant, countries. Moreover, the true burden of cancer in Libya cannot be reliability determined. The lack of a national cancer registry, constantly shifting demographics, and incomplete records of patients with cancer leads to biased calculations of any cancer-related epidemiological parameter (12, 13); all of which were further amplified by the breakout of the Libyan civil leading to further cancer disparities (14). The most recent report on Libyan cancer statistics, based on the Misurata City Cancer Registry, describe a crude incidence rate of 71.1 per 100,000 with breast, colorectal, and lung cancer being the most prevalent cancers (12). A 2015 report, based on the Benghazi City Cancer Registry, documented a world age-standardized incidence of all site cancers were 135.4 and 107.1 per 100,000 for males and females, respectively (14). Moreover, lung and breast cancers were the most prevalent cancers among males and females, respectively. Nonetheless, epidemiological cancer statistics with regards to Libya lack generalizability due to the reasons mentioned above.

Jordan, an upper middle-income country with a GDP of 28.8 billion, provides cancer care to all of its citizens at no costs through public hospitals, university hospitals, and the King Hussein Cancer Center (KHCC) (15). Jordan’s cancer care primarily targets the treatment element of the cancer care continuum as it does not have a national control policy or subsidized large scale prevention programs. Moreover, in terms of screening, Jordan’s only active and institutionalized program is its breast screen program (16). Other programs pertaining to colorectal cancer and cervical cancer among others are extremely limited and have no associated databases. KHCC is Jordan’s only specialized cancer care center and is a leading comprehensive center within the region (17). KHCC provides cancer care for patients from neighboring countries, especially those under difficult humanitarian conditions. Such countries include Iraq, Yemen, Syria, Sudan, Libya, Lebanon, and Palestine. The numbers of Libyan patients presenting to KHCC has significantly increased since the 2011 civil war. This was further boosted by an official agreement signed between KHCC and the Libyan Ministry of Health in 2017 by which the Libyan government aimed to treat its cancer patients within specialized centers by covering its costs at KHCC. However, such an agreement fell short in 2021 due to increasing and unpaid debts on the Ministry’s behalf.

Due to the gap in the literature with regards to cancer epidemiology of displaced Libyan patients, we aimed to demonstrate the epidemiology of Libyan cancer patients who received treatment over more than a decade at KHCC, including the most common types of diagnoses, the stages at presentations at KHCC, and the treatment modalities received. Moreover, we aimed to provide concerned bodies with a number of relevant recommendations for potential use for future patient recruitment, treatment, and follow-up.

## 2 Methods

Relevant data on all Libyan patients with cancer previously presented to/treated at KHCC from 2006 to 2019 was extracted from the Center's Cancer Registry. The Registry was established in 2006 and is an electronic database that involves the demographic, clinical, and psychosocial data of any and all patients presenting to KHCC. Extracted variables included age at diagnosis, date of first contact with KHCC, definitive diagnosis, tumor details (e.g., site of tumor and histology), and treatment details. Stage at presentation was collected according to the American Joint Committee on Cancer (AJCC) TNM system and Surveillance, Epidemiology, and End Results (SEER) summary staging system. The 6^th^, 7^th^ and 8^th^ editions of the TNM systems were used, according to the time period at which the data were collected by the Registry. For the SEER summary staging system, cases before 2018 were stratified based on the 2000 edition, while the 2018 edition was effective on January 1st, 2018 onwards. For the purpose of comparison, data regarding SEER stage 7 for Jordanian cancer patients during the same time period, in terms of age, gender, anatomical site of disease, and pathological diagnosis were also collected. Categories of the 2000 and 2018 versions of the SEER summary stage are illustrated in **Table 1** (18, 19). The aforementioned data was provided by the KHCC Cancer Registry manager, who was anonymized to the purposes of the study.

**Table 1:** Description of different categories used in the 2000 and 2018 versions of the SEER summary stage.

Code	Definitions	
	2000 version	2018 version
0	In situ	In situ
1	Localized only	Localized only
2	Regional by direct extension only	Regional by direct extension only
3	Regional lymph nodes involved only	Regional lymph nodes only
4	Regional by both direct extension and lymph node involvement	Regional by both direct extension and lymph node involvement
5	Regional, NOS (Not Otherwise Specified)	*Can no longer be coded
7	Distant site(s)/node(s) involved	Distant site(s)/node(s) involved
8	Benign/borderline	Benign/borderline
9	Unknown if extension or metastasis (unstaged, unknown, or unspecified) Death certificate only case	Unknown if extension or metastasis (unstaged, unknown, or unspecified) Death certificate only case

*Code 5 (regional, NOS) in the 2000 version can no longer be used in the 2018 version.

The resultant data was cleaned, re-organized, and re-categorized using Microsoft Excel. Data were later handled using SPSS version 23. Categorical variables were presented as frequencies [n(%)], while continuous variables were presented as means and their associated standard deviations. Data on cancer frequency and staging among patients were stratified by age (adults vs. pediatrics), biological sex (male vs. female), and nationality (Libyan vs. Jordanian). Any variable with more than 10% missing data was removed from the final analysis.

## 3 Results

### 3.1 Demographic details

**Figure 1:**
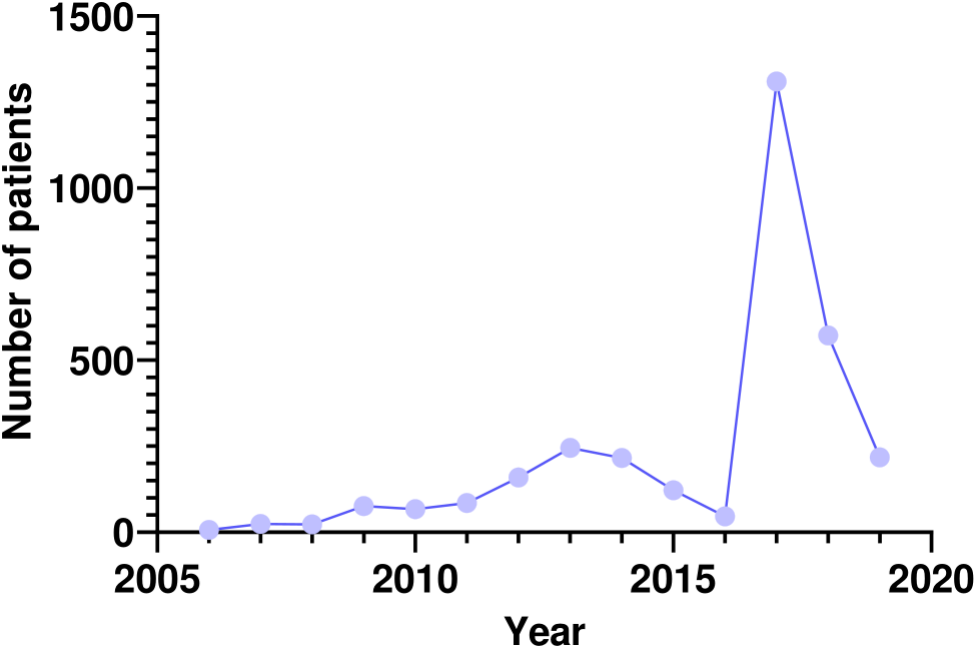
Trend of referral of Libyan patients with cancer between 2006 and 2019. Number of cases (n=3170) had significantly increased after the first the Libyan civil war in 2011. A surge of cases coincided with the Libyan Ministry of Health and KHCC treatment agreement signed in 2017.

**Figure 2:**
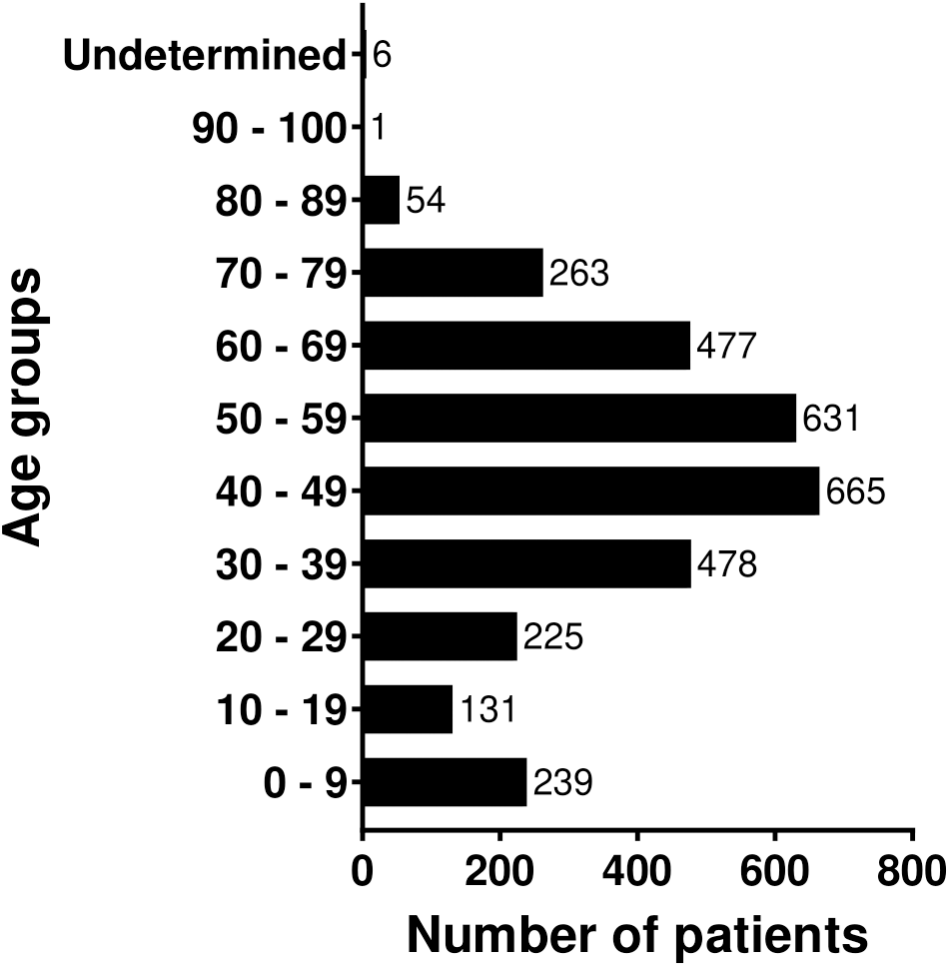
Libyan patients with cancer age distribution.

A total of 3170 Libyan cancer patients were treated at KHCC between June 2006 and 2019. After the beginning of the political and military conflict in Libya in 2011, the number of Libyan patients increased significantly, with 2888 (91%) cases recorded between 2012 and 2019. In June 2017, an agreement was signed with the Libyan Ministry of Health to provide health care to Libyan cancer patients. This agreement was associated with a surge in the number of cases (n=1310, 41.3%). Since then, KHCC has treated 2100 patients, representing 66% of the total patients’ number (**Figure 1**). For the whole group, there was an almost equal representation of males and females, with a ratio of 1:1.2 including 1445 male (45.6%) and 1725 female (54.4%) patients. The patients` age range was wide spanning newborns up to 93 years. The mean and median ages were 45 and 31.5 years, respectively. The patients were predominantly adults, with 71% (n=2251) ranging between 30 and 69 years. Only 355 patients (11%) fell into the pediatric age group. Age group distribution is illustrated in **Figure 2**.

### 3.2 Classification according to site of tumor origin

Tumors originating from the breast, hematolymphoid (HL) and gastrointestinal tract (GIT) were the most common, constituting 54.2% (n=1719) of all cases collectively, including 21% (n=665), 17% (n=539) and 16.2% (n=515), of all cases respectively. Tumors in other locations constituted less than 10% each of the total number of cases. **Table 2** lists the details of the location of tumors among Libyan cancer patients treated at KHCC.

**Table 2:** Organ/system-based classification of all cases.

Organ/System	Number of cases (%)
Breast	665 (21%)
Hematolymphoid system	539 (17%)
Gastrointestinal tract	515 (16.2%)
Central nervous system	273 (8.6%)
Female genital tract	182 (5.7%)
Head and neck	166 (5.2%)
Lung	149 (4.7%)
Soft tissue	131 (4.1%)
Endocrine system	129 (4%)
Male genital tract	122 (3.8%)
Urinary system	95 (3%)
Pancreatobiliary system	76 (2.5%)
Bone	51 (1.7%)
Unknown primary	23 (0.7%)
Skin	23 (0.7%)
Liver	18 (0.6%)
Mediastinum	8 (0.3%)
Mesothelium	5 (0.2%)
Total	3170 (100%)

### 3.3 Classification according to age and gender

#### 3.3.1 Adult female cancer patients

In total, there were 1570 patients, with a mean and median age of 50.1 and 47 years, respectively. Breast tumors were the most common cancer in females (n=656, 41.7%), followed by GIT tumors (n=222, 14.1%), with a median age of 45 and 52 years, respectively. Tumors originating from the female genital tract accounted for 11.2% (n=177) of all tumors; almost half (n=84, 5.3%) of which affecting the endometrium in post-menopausal women (median age of 54 years). Interestingly, tumors of the endocrine system were more common in females (n=75, 4.7%), two-times the incidence of those in males (n=34, 2.7%). This was primarily a reflection of thyroid gland (n=48, 3%), and pituitary gland tumors (n=16, 1%). The median age for thyroid and pituitary gland tumors were 38 and 30 years, respectively, which was younger than the median age for all other types of malignancy.

#### 3.3.2 Adult male cancer patients

**Figure 3:**
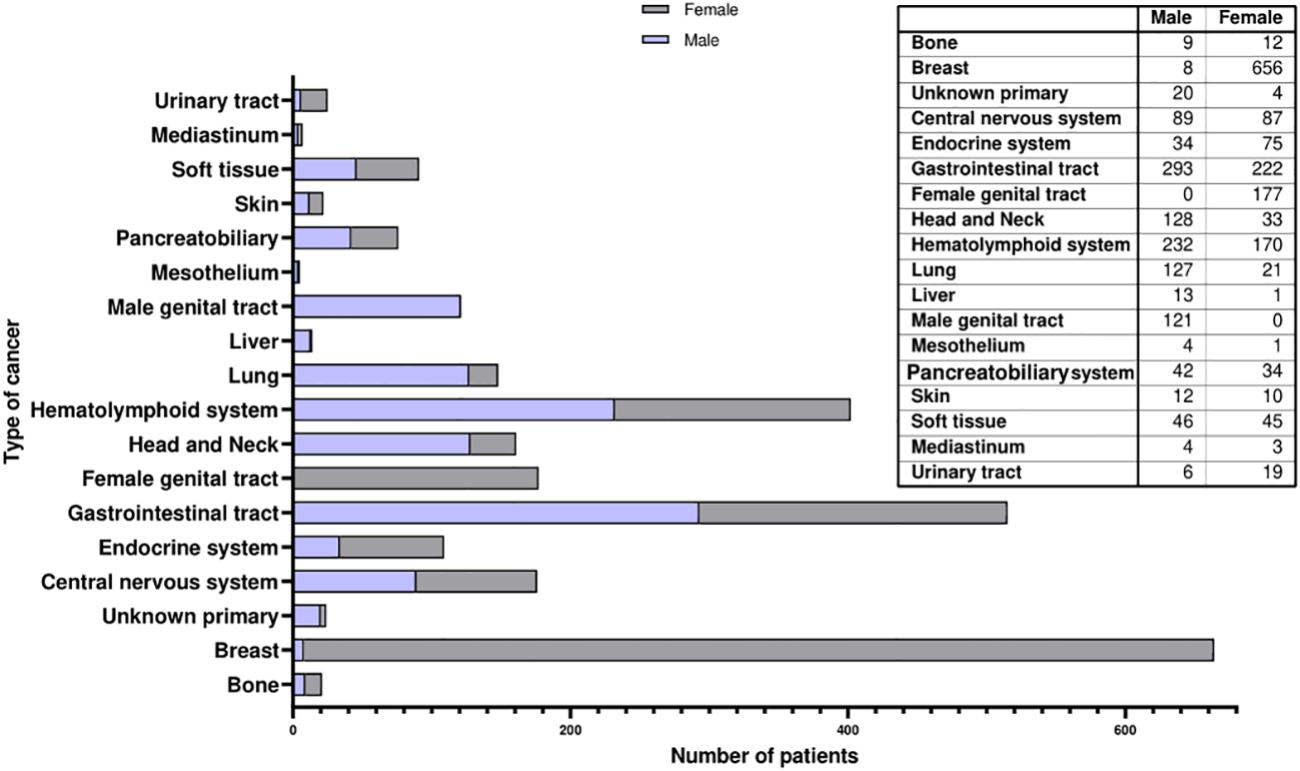
Gender-based stratification of adult tumors.

In total, there were 1245 patients, with a mean and median age of 54.6 and 55 years, respectively. Gastrointestinal tract tumors were the most common among male patients (n=293, 23.6%), followed by HL malignancies (n=232, 18.7%). Lung carcinoma (n=127, 10.2%) and head and neck tumors (n=128, 10.3%) were the 3^rd^ and 4^th^ most common tumors among Libyan adult males. This prevalence is higher than what is reported for adult Libyan females for lung carcinoma and head and neck tumors (n=21, 1.3%, and n=33, 2.1%, respectively). The median age for GIT, HL, Lung and head and neck tumors was 56, 43, 61, and 52 years, respectively. Tumors of the male genital tract ranked 5^th^ (n=121, 9.7%) predominated by prostate cancer (n=108, 8.6%), mostly in elderly patients (median age of 66 years). **Table 3** and **Figure 3** depicts the distribution of cancer cases according to gender.

**Table 3:** Gender-based stratification of adult tumors.

System/Organ	Number	Male (%)	Female (%)
Bone	21	9 (0.7%)	12 (0.7%)
Breast	664	8 (0.6%)	656 (41.7%)
Unknown primary	24	20 (1.5%)	4 (0.2%)
Central nervous system	176	89 (7.1%)	87 (5.6%)
Endocrine system	109	34 (2.7%)	75 (4.7%)
Gastrointestinal tract	515	293 (23.6%)	222 (14.1%)
Female genital tract	177	–	177 (11.2%)
Head and neck	161	128 (10.3%)	33 (2.1%)
Hematolymphoid system	402	232 (18.7%)	170 (10.8%)
Lung	148	127 (10.2%)	21 (1.3%)
Liver	14	13 (1.0%)	1 (0.1%)
Male genital tract	121	121 (9.7%)	–
Mesothelium	5	4 (0.3%)	1 (0.1%)
Pancreatobiliary system	76	42 (3.5%)	34 (2.3%)
Skin	22	12 (1.0%)	10 (0.7%)
Soft tissue	91	46 (3.7%)	45 (2.9%)
Mediastinum	7	4 (0.3%)	3 (0.2%)
Urinary system	83	64 (5.1%)	19 (1.3%)
Total	2816	1246	1570

#### 3.3.3 Pediatric cancer patients

In total, there were 355 cases, with a mean and median age of 6.8 and 5.0 years, respectively. Hematolymphoid tumors (n=137, 38.5%) were the most common among the pediatric age group. Acute leukemia represented around one quarter of cases (n=78, 22%), with a median age of 5 years. Central nervous system (CNS) tumors accounted for 19.5% of tumors among pediatric patients (n=69); a third of which (n=32, 9%) were infratentorial tumors mainly medulloblastoma (n=13, 3.6%) with a median age of 3 years. Also, 28 (7.8%) cases of eye tumors were predominated by retinoblastoma (n=26, 7.3%) with a mean age of 8 months and a median age of 1 year. Other tumors included bone tumors (n=30, 8.5%), and Wilms’ tumor (n=12, 3.4%). Also, there were 20 (5.6%) cases of neuroendocrine tumors, with a predominance of neuroblastoma (n=16, 4.5%, median age 2 years). As a group, germ cell tumors were encountered in 6 cases (1.6% of the total malignant tumors in pediatrics), including 4 cases of ovarian tumors in females, and 2 cases of testicular and mediastinal germ cell tumors in male patients. Two cases of hepatoblastoma and malignant rhabdoid tumors, each were encountered. A single case of pleuropulmonary blastoma in a 2-year-old boy and a single case of cutaneous malignant melanoma were also diagnosed. A summary of pediatric tumors is presented in **Table 4**.

**Table 4:** Frequency of site-specific tumors among pediatric Libyan patients treated at KHCC between 2006 and 2019.

System/Organ	Number of cases (%)
Hematolymphoid system	137 (38.5%)
Central nervous system	69 (19.5%)
Soft tissue	40 (11.3%)
Bone	30 (8.5%)
Eye	28 (7.8%)
Endocrine system	20 (5.6%)
Urinary tract	12 (3.4%)
Head and neck	5 (1.4%)
Female genital tract	5 (1.4%)
Liver	4 (1.1%)
Breast	1 (0.3%)
Lung	1 (0.3%)
Male genital tract	1 (0.3%)
Skin	1 (0.3%)
Mediastinum	1 (0.3%)
Total	355 (100%)

### 3.4 Classification according to stage of presentation at KHCC

**Figure 4:**
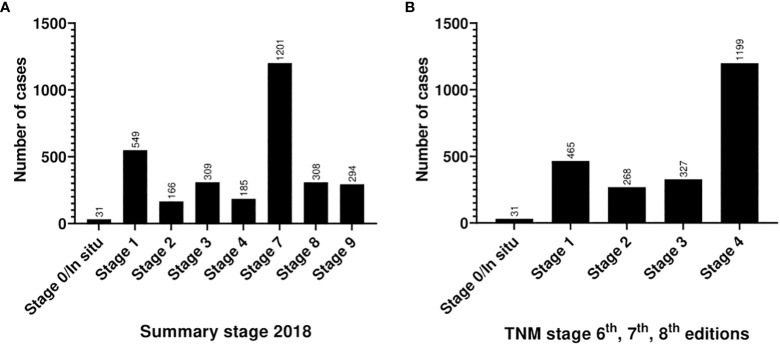
Stage-wise stratification of patients according to **(A)** the 2018 version of Summary Stage and **(B)** the 6^th^, 7^th^ and 8^th^ editions of the AJCC TNM staging system.

According to the 2018 version of SEER (**Table 1**) 37.8% (n=1201) of patients presented with distant metastasis (Stage 7; consistent with metastatic tumor), another 38% (n=1240) were distributed between stages 0–4. Based on the 6^th^, 7^th^ and 8^th^ editions of the AJCC TNM staging system, around half of cases presented with advanced stage at first encounter at KHCC; namely stage 4 (n=1199, 37.8%) and stage 3 (n=325, 10.2%). The exact TNM stage was not assigned for 873 patients (27.5%). **Figure 4** displays further analysis. Among SEER stage 7 category, the most common age group affected was 40-59 years (n=475, 39.5%) and was equally distributed between male and female patients. Almost all (n=1189, 99%) were previously diagnosed prior to visiting KHCC, and around one-third (n=426, 35.4%) have already received different modalities of treatment/care, including palliative care (n=365, 30.4%). Lung represented the most common diagnosis (73%, n=109 out of a total of 194 cases) in this group of patients, followed by the GIT (41%, n=212 out of a total of 515 cases), female genital tract (25.8%, n=47 out of a total of 182 cases), and breast (25%, n=157 out of a total of 620 cases). In comparison, SEER stage 7 Jordanian cancer patients at the same period represented only 18.5% (n=5704) out of a total of 30709 cases. The overall distribution of cases is comparable between Libyan and Jordanian patients, with minor discrepancies. In **Table 5**, the proportion of SEER stage 7 among the two populations is significantly different. For example, in general, breast cancer cases constitute 21% (n=665) and 21.5% (n=6632) of Libyan and Jordanian patients, respectively. Unfortunately, 25% (n=157) of the Libyan breast cancer patients were seen for the first time at KHCC with metastatic disease, compared to 18.8% (n=1249) of their Jordanian counterparts. This applies also to female genital tract tumors, as Libyan patients were 2.5 times more likely to present as SEER stage 7 compared to Jordanian patients.

**Table 5:** Comparison of between Jordanian and Libyan patients with cancer in terms of cases presenting with SEER stage 7 advanced cancer.

System/Organ	Libyan		Jordanian	
	All patients (n=3170)	SEER 7 patients (n=1201; 37.8%)	All patients (n=30709)	SEER 7 patients (n=5704; 18.5%)
Breast	665 (21%)	157 (25%)	6632 (21.5%)	1249 (18.8%)
Gastrointestinal tract	515 (16.2%)	212 (41%)	6810 (22.1%)	1098 (16.1%)
Lung	149 (4.7%)	109 (73%)	2430 (7.9%)	1078 (44.3%)
Female genital tract	182 (5.7%)	47 (25.8%)	6810 (7.3%)	211 (9.3%)

### 3.5 Classification according to treatment modalities received at KHCC

**Figure 5:**
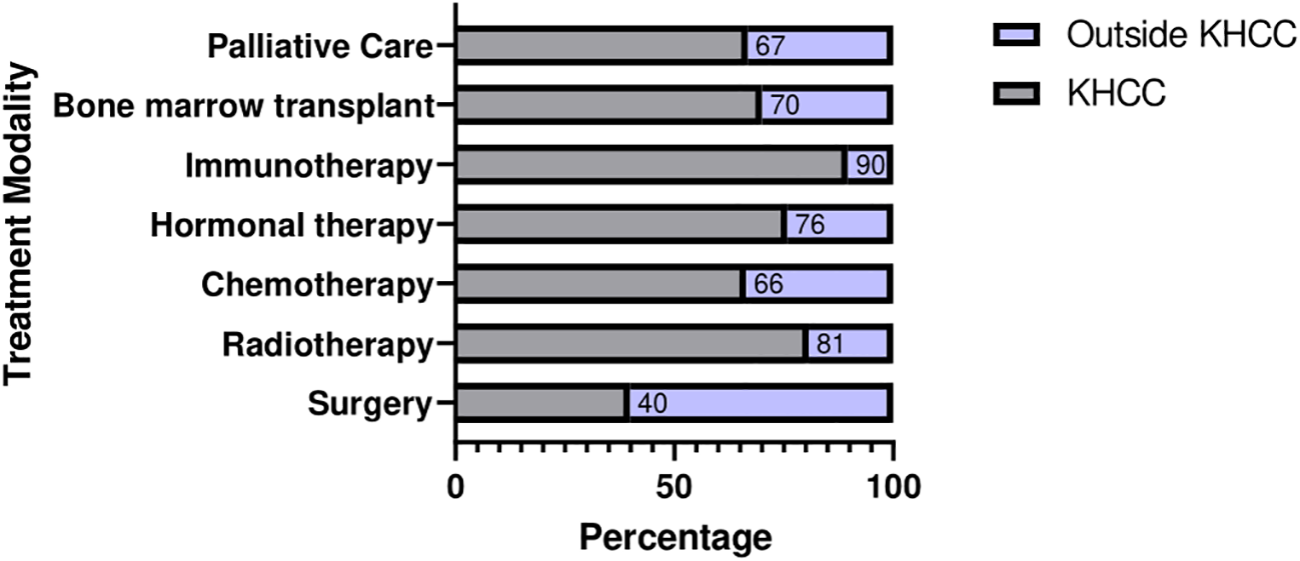
Comparison of treatment options offered to patients before and after referral to KHCC.

At first encounter at KHCC, almost all cases (n=3046, 96%) were previously diagnosed, and a third (n=1079, 34%) received previous treatment, either at their home country “Libya” (n=1908, 60.1%) or in other neighboring countries including Jordan (outside KHCC), Tunisia, and Egypt among others. Only 3.7% (n=117) and 19% (n=600) cases were first diagnosed and treated at KHCC, respectively. King Hussein Cancer Center offered a wide range of treatment options according to the most recent national and international guidelines and based on multidisciplinary clinics’ decisions. Out of the 3170 cases, 15.7% (n=500) underwent different types of surgical interventions. Radiotherapy and chemotherapy were offered to 19% (n=608) and 28.8% (n=913) of all patients, respectively. A minority of patients received hormonal therapy (n= 229, 7.2%), immunotherapy (n=26, 0.8%), and bone marrow transplant (n=35, 1.1%). Unfortunately, 9.8% (n=312) patients were candidates for palliative care, and another 4.8% (n=155) have already been receiving palliative care at first presentation to KHCC, all of whom (n=467) were registered after the 2017 agreement. Treatment options are summarized in **Figure 5**.

### 3.6 The three most common types of malignant tumors in Libyan patients treated at KHCC

#### 3.6.1 Breast

A total of 665 cases of breast tumors presented to KHCC with the vast majority being malignant (n=620, 93.3%), predominantly in females (n=612, 98.7%), with eight (1.2%) male patients. For malignant tumors, the age ranges between 24 and 82 years, with a mean and median of 47 and 45 years, respectively. Almost all cases (n=634, 95%) were diagnosed and 43.0% (n=287) treated prior to referral to KHCC. One-fourth of patients (n=157, 25%) had distant metastasis (SEER 7; AJCC TNM stage 4) at presentation to KHCC. Radiotherapy, hormonal therapy, and chemotherapy were given to 24.0% (n=150), 30% (n=187), and 31.5% (n=195) of all patients, respectively. Moreover, 79 patients (11.8%) received palliative care either before or after referral to KHCC.

#### 3.6.2 Hematolymphoid system

There were 539 patients with HL malignancy, with a mean and median age of 34 years with a male to female ratio of 2:1. Collectively, Non-Hodgkin lymphoma (NHL) (n=163, 30.3%) and acute leukemia (n=152, 28.2%) represented more than half of cases. The vast majority of patients (n=529, 98%) were previously diagnosed, and around one-third (n=163, 30%) received treatment before referral to KHCC. At KHCC, chemotherapy, radiotherapy, and bone marrow transplant were offered to 40.8%, (n=220), 10.2% (n=55), and 6.1% (n=33) of all patients, respectively. Seventy-eight patients (14.5%) received palliative care either before or after referral to KHCC.

#### 3.6.3 Gastrointestinal tract

A total of 515 patients with GIT tumors presented to KHCC, including 82.0% (n=422) cases with malignant tumors, predominated by colon (n=412, 80%), followed by stomach (n=85, 16.5%). More than half of the cases affected males (n=294, 57%). The patients` age ranged between 20 and 87 years, with a median age of 54 years. The vast majority of cases were diagnosed (n=485, 94%) and many received treatment (n=192, 37%) before referral to KHCC. Forty-one percent (n=212) had distant metastasis (SEER7; AJCC TNM stage 4) at first presentation to KHCC. Twenty percent (n=104), 28.7% (n=148), and 9.5% (n=49) had surgery, received chemotherapy, and radiotherapy at KHCC. Palliative care was offered to 20.5% (n=104) of all patients with GIT tumors.

## 4 Discussion

Cancer is considered a leading cause of morbidity and mortality worldwide, regardless of the level of socioeconomic status (20). Under harsh humanitarian conditions, cancer diagnosis and management become more challenging. Cancer patients from areas of conflict; the Middle East as an example, often get diagnosed at late stages leading to poor outcome. This is probably due to the poor living conditions, lack of screening programs and awareness campaigns, limited access to care, and scarcity of the available resources. Even when they attempt to seek medical advice outside their home countries, they are unfamiliar with the newly encountered healthcare systems (21). By tracking the numbers of Libyan patients referred to KHCC over the last fourteen years, a steady increase is noted, especially after the Libyan political crisis in 2011, and the subsequent years of political turmoil. This has reflected negatively on all aspects of civil lives including insecurity and economic instability, in a manner that led to the flaccidity of the Libyan healthcare system.

According to GLOBOCAN’s 2020 cancer statistics, the estimated world age-standardized incidence rate for all cancers in Libya is 185.4 per 100,000 people. The crude incidence rate of all cancers among Libyans is 209.8 per 100,000 people. Based on the report, the most common malignancies among the Libyan female population are breast cancer (47.3 per 100,000), colorectal cancer (15.6 per 100,000), cervical cancer (14.4 per 100,000), and lung cancer (13.7 per 100,000). In contrast, prostate cancer (30.6 per 100,000), lung cancer (30.0 per 100,000), colorectal cancer (22.4 per 100,000), and stomach cancer (15.0 per 100,000) are the most prevalent among Libyan males. These numbers are significantly higher to what was reported by the Benghazi Cancer Registry (14), which further accentuates the fact that single city registries underestimate the true burden of cancer. Particularly, the Benghazi Cancer Registry, which is the only source that provides comparable statistics to GLOBOCAN 2020, underestimates the incidence of prostate cancer (14.8 per 100,000) among males and lung (3.1 per 100,000) and cervical cancer (4.6 per 100,000) among females.

According to our data, breast remained the most common cancer referred to KHCC and the most common cancer in females, in alignment with the aforementioned statistics. Among adult Libyan males with cancer, lung cancer and prostate cancer ranked as the 3^rd^ and 5^th^ most common tumors referred to KHCC. Under-representation of prostate cancer in our cohort might be related to the general good outcome of treatment by hormonal therapies which might not demand referral abroad. Per the latest GLOBOCAN estimates of 2020, lung cancer is the 2^nd^ most common diagnosed cancer among both sexes. It is the most common diagnosed cancer in males and the 3^rd^ most common in females (22). Interestingly, referrals of lung cancer among our Libyan population are underwhelming as it ranks 7^th^ among the whole group, 4^th^ among males, and 10^th^ among females. The poor representation of lung cancer among referred Libyan patients could be attributed to the complex paradigms affecting patient referral. Such factors include poor functional status, rapid deterioration of clinical status, patient-oriented factors, age, and lack of symptoms (23). Other factors influencing referral are the lack of awareness of primary physicians with systemic therapies for advanced lung cancer or the financial constrictions within which these cases fall. The latter is more relevant to our patients since many Libyan patients with cancer are dependent on governmental funding to be able to afford systemic therapy at KHCC.

While Libyan patients represent around 10% of treated Jordanian patients’ volume, they are twice as likely to present with advanced stages. The nature of such presentations, in addition to the increasing volume of patients will most likely strain the oncology medical services provided within KHCC and across Jordan. Additionally, the costs of treatment will further overwhelm the healthcare infrastructure of a country with already limited resources. This was clearly demonstrated throughout the Syrian crisis as it costed King Hussein Cancer Foundation Goodwill Fund a total of around 11,400,000 million U.S. dollars to cover the treatment of only 356 Syrian patients with cancer (17). Using such data, it is estimated that the annual costs of treating Syrian patients with cancer is around 22,111,118 million U.S. dollars. Unfortunately, the financial strains of this ever-increasing volume of patients manifested as the cancellation of the governmental treatment agreement between the Libyan Ministry of Health and KHCC. Such was the case due to the rising amounts of debt held by the Libyan authorities which subjected their beneficiaries within Jordan to cancer care inaccessibility.

There are a few limitations encountered in the study. Such include the use of two different SEER summary staging systems for the classification of cases; 2000 and 2018 summary staging system. This has created some discrepancy as SEER stage 5 was used in the 2000 manual but is no longer used in the updated 2018 classification system (18, 19). Thus, it was not possible to reclassify patients registered in this group (n=137, 4.3%) using updated guidelines. Furthermore, a significant proportion of patients were diagnosed and treated before referral to KHCC; thus, documentation of their previous pathological diagnosis, laboratory tests, treatment plans, and follow-up visits was suboptimal and of which impact could not be reflected within the study. Also, outcome variables such as overall survival and recurrence free survival were not demonstrated for Libyan patients. Such is the limitation of the KHCC Cancer Registry as it only provides follow-up for international patients up until 6 months of their first contact with KHCC. Extracting such information, even within the sole context of this study, remains difficult as patients traveling back to Libya lose follow up due to a variety of reasons including travel and financial difficulties, and the volatile security and economic conditions.

### 4.1 Recommendations

Based on the presented data, we could draw on a number of recommendations. In absence of a well-established national Libyan cancer registry (12), the results of this study might lay the groundwork for the drafting and development of a cancer care strategy for the Libyan populace and their associated displaced populations. Both the literature and KHCC’s data demonstrated that breast and GIT cancers were among the most prevalent cancers affecting this vulnerable population (22, 24). However, screening activities and their associated scale are extremely limited within Libya. This had predisposed patients to late diagnoses and presenting in advanced stages (25, 26). Therefore, the establishment of nationwide screening programs will aid in the early detection of cases which will reduce cancer burden and its costs for both affected individuals and the concerned authorities. Such programs should complement the already successful programs within big Libyan cities such as Tripoli and Benghazi. Moreover, the initiation of efforts within larger, more populated cities would cut off the costs of raising new capital through the pre-existing programs and facilities.

Screening programs should be complemented by simple cost-effective measures as to increase their effectiveness and reach within the population. Awareness campaigns for cervical cancer were already launched in many Libyan cities as of 2020 and adopted a digital format as of 2021 (24). Such campaigns must be sustained and extended to other prevalent cancers. It is documented that patients, especially those within conservative communities, may refrain from pursuing cancer care or diagnosis due to social stigma (e.g., cancer diagnosis may lead to divorce among families) (9). KHCC’s experience in leading the “Jordanian Breast Cancer Program” can be emulated as a proof-of-concept model.

It appears that patients referred to KHCC for palliative care demand better triaging of advanced cases. While the palliative care track does exist within the Libyan healthcare system, it faces a significant shortage across the entire country. Libya’s National Cancer Control Program is trying to address such an issue (24). Therefore, it is important to devise a referral strategy for palliative care patients that takes into account the emotional, psychological, and financial burdens associated with this vulnerable group.

Taking advantage of the overlapping culture, language, demographics and risk factors of the Libyan and Jordanian populations, the success and resilience of the KHCC model can be adopted within Libya through a satellite center (27–29). This can help to establish an optimum solution to the challenges facing cancer management in Libya, through augmentation of the Libyan oncology healthcare service to provide a more convenient atmosphere for patients, as they will be treated among their families, leading to a better psychosocial support, and subsequently reserve outside referral only for those who need complex and major interventions.

The implementation of twining programs (30) and telemedicine oncology services (28, 31) as means to discuss cases has revolutionized the management of rare and difficult cases at KHCC. A “twining program” refers to the extended and long-term collaboration and sharing of skills and experience between an experienced cancer center in an upper-income country with a cancer program in a low- or middle-income country (32). While most twinning programs involve personnel exchanges between different centers, others utilize telemedicine as a medium of facilitating such interactions in the aim of evolving patient care (27). KHCC’s twinning program with Canada’s Hospital for Sick Children was established in 2003 and has proved effective in augmenting the multidisciplinary care of pediatric neuro-oncology cases (30). Twining, through videoconferencing, is a cost-effective tool that is able to provide input on treatment, improve case approach, educate personnel, and introduce novel concepts of cancer care. Similarly, the twinning program between KHCC and Saint Jude’s Children’s Hospital, which was established in 2003, proved the effectiveness of such endeavor as outcomes of patients with retinoblastoma equaled that of developed nations (31).

Twining programs can be supported with the establishment of multidisciplinary clinics in which cancer experts from all fields of the cancer care continuum meet to discuss the most optimal approach for complex cases. In addition to their ease of implementation, these programs have proved their efficacy in improving patient outcomes, particularly mortality rate (33). Multidisciplinary programs were shown to be cost effective within an oncological context. The implementation of such boards for breast cancer in Mozambique and for lung cancer at the Kingston Health Sciences Center improved both outcomes and reduced costs (34, 35). Such reduction is attributed to decreased patients’ visits, better planning and administration of treatment, improved clinical course, and more efficient resource utilization. If cemented across Libya and its neighboring countries, these efforts could also enable the accurate monitoring of patients’ follow-up data and survival status.

That being said, any and all policies that may be discussed among Libyan concerned bodies and cancer care authorities must base its priorities on a strong ground of epidemiological data. Therefore, the creation, maintenance, and constant expansion of a national comprehensive cancer registry is vital in the understanding of the burden of cancer among the Libyan populace. The lack of such reliable data can be sensed within the literature as only a handful of studies attempted to deconstruct their respective population-based/center-based registers, of which results had shown significant underestimation compared to international figures. With regards to such a case, the Jordanian experience might be a suitable model as its cancer registry, Jordan Cancer Registry, is considered a well-established database of high international standards.

### 4.2 Conclusion

Treatment of Libyan patients with cancer requires high quality services necessitating collaboration of the various governmental and private sectors. The cooperation between KHCC and the Libyan Ministry of Health facilitated the proper treatment of Libyan patients with cancer. However, war and ongoing conflicts still emanate a direct negative impact on the survival outcomes of Libyan cancer patients. Therefore, the drafting and implementation of large, long-term policies in collaboration with experts and concerned bodies, which target the facilitation of cancer care to such vulnerable populations, are essential to the improvement of their outcomes and overall wellbeing.

## Data availability statement

All data/data sets associated with this project will be made available by the corresponding author upon reasonable request.

## Ethics statement

The research was approved by the Institutional Review Board (IRB) at King Hussein Cancer Center under number 20KHCC66. The request for Waiver of Informed Consent was granted by KHCC- IRB, since the study, involves collection of existing data from the electronic medical records and the Center’s Cancer Registry, with no direct interaction with participants.

## Author contributions

Conceptualization: MA-H and AM; Methodology: ME; Formal analysis: ME and AA; Writing – Original Draft: MA-H, AM, and ME; Writing – Review & Editing: MA-H and AA; Supervision: MA-H and AM. All authors contributed to the article and approved the submitted version.
